# Authors’ Reply: Advancing Digital Health Integration in Oncology

**DOI:** 10.2196/72477

**Published:** 2025-03-07

**Authors:** Yura Lee, Ye-Eun Park

**Affiliations:** 1 Department of Information Medicine Asan Medical Center University of Ulsan College of Medicine Seoul Republic of Korea

**Keywords:** mHealth, user experience, cancer, technology acceptance model, structural equation modeling, health care app, mixed-method study, medical care, digital health care, cancer survivors, disparities, health status, behavioral intervention, clinician

We appreciate the in-depth review and the critical perspectives provided on our study, “User Experience and Extended Technology Acceptance Model in Commercial Health Care App Usage Among Patients With Cancer: Mixed Methods Study” [1]. This reply aims to address and expand upon the discussion points raised in the letter [2], particularly those concerning the broader implications of telehealth and digital health solutions.

The point raised by Umar Khan and Tariq [2] about enhancing telehealth solutions to address accessibility issues is pivotal. Our study, while primarily focusing on user engagement with health care apps among cancer survivors, indirectly highlighted the urban-rural access disparities and digital literacy gaps. These findings suggest an urgent need for comprehensive telehealth infrastructure that is sensitive to regional disparities. Our data indicated a higher usage rate of health care apps among urban dwellers, likely due to better access to digital resources. This observation underscores the importance of policy interventions aimed at improving digital literacy among older and rural populations, which could significantly bolster the effectiveness of telehealth solutions [3].

Furthermore, the necessity of both patient and provider acceptance for sustained digital health engagement was a key finding in our research. Our study underscored the critical role of clinician endorsements as external motivators that significantly influence both the intention to use and the actual usage of health care apps. However, the real-world application of digital tools in clinical settings often reveals a gap between controlled study environments and everyday clinical practice. In addressing this, we aimed to minimize such discrepancies by using commercial apps without structured reminders, thus fostering more natural user engagement.

The suggestion to integrate robust patient portal features aligns well with our findings on the importance of user-friendly interfaces and perceived utility in promoting app engagement. While our study did not specifically investigate patient portals, incorporating such features could enhance patient autonomy by facilitating access to personalized health information and improving communication with health care providers. To further enhance patient engagement, integrating approaches such as shared decision-making and co-design into digital health tools would be beneficial, fostering a user-centered design that accommodates patient preferences and needs [4,5].

Despite its focus on patients with cancer, our study’s application of structural equation modeling to identify key factors influencing digital health acceptance is significant, particularly as patient-physician joint decision-making is crucial for this patient group. We also acknowledge the call for further research to assess the generalizability of our findings. Our study’s cohort, primarily sourced from tertiary referral hospitals in South Korea, may not fully represent the broader population of cancer survivors. This limitation, coupled with South Korea’s unique health care context, may affect the external validity of our results. Future studies should aim to include a more diverse participant pool to provide insights that are applicable on a global scale.

In conclusion, we are thankful for the thoughtful critique and the opportunity to engage in this enriching dialogue, which has reinforced the importance of a multidisciplinary approach in advancing digital health care.
